# Revolutionizing healthcare: Unleashing the power of regulatory measures to enforce smoke-free policies in Indonesian medical facilities

**DOI:** 10.34172/hpp.025.43146

**Published:** 2025-11-04

**Authors:** Muhamad Ridwan, Dwi Noerjoedianto, Muhammad Syukrii, Abdilah Ahsan

**Affiliations:** ^1^Public Health Sciences Study Program, Faculty of Medicine and Health Sciences, Universitas Jambi, Muaro Jambi, Indonesia; ^2^Department of Economics, Faculty of Economics and Business, University of Indonesia, Jakarta, Indonesia

**Keywords:** Cigarette smoking, Community health centers, Smoke-free policy, Tobacco control

## Abstract

**Background:**

The global challenge of tobacco usage poses significant hurdles in policy implementation. Local governments’ smoke-free policies have struggled with optimal enforcement, hampering effective smoking control. This study evaluates smoke-free zone implementation in healthcare services within Muaro Jambi, Indonesia.

**Methods:**

This convergent mixed-methods study collected data from June to December 2023. The quantitative phase entailed a spatial survey across 74 healthcare facilities (hospitals, community health centers, and clinics), facilitated by the KoboToolbox application. Data were analyzed using SPSS 16.0 for descriptive statistics and QGIS 3.30.2 for spatial mapping. The qualitative component employed content analysis of in-depth interviews with 31 purposively selected informants representing healthcare facilities and policymakers. Interviews were conducted by trained enumerators, transcribed verbatim, and analyzed using conventional content analysis techniques following Bengtsson’s methodology.

**Results:**

Most healthcare facilities (74.3%, 95% CI: 62.8-83.8%) failed to implement smoke-free areas effectively, with hospitals and community health centers showing 100% non-compliance and clinics at 61.2%. Key barriers included inconsistent policy socialization, inadequate leadership enforcement, absence of sanctions, and cultural acceptance of smoking in outdoor areas. Effective implementation correlated with strong leadership commitment and consistent rule enforcement.

**Conclusion:**

The effectiveness of smoke-free policies in Indonesian healthcare facilities hinges on robust regulatory measures and consistent oversight from local governments. Leaders and staff serve as role models, ensuring compliance through their actions. While regional regulations are critical for tobacco control, their success depends on unwavering support from local and organizational leaders, emphasizing the need for active involvement from decision-makers and stakeholders.

## Introduction

 The pervasive global issue of cigarette consumption has prompted sustained scholarly attention. Robust efforts, reflected in diverse initiatives and smoking prohibitions, aim to mitigate the prevalence of smokers. Recognizing smoking as a substantial health challenge is crucial on a global scale, as emphasized by Dai et al.^1^ The World Health Organization (WHO) report reveals a staggering global count of approximately 1.1 billion smokers.^2^ Insights from Basic Health Research in 2018 further accentuate the concern, showing an escalation in smoking prevalence among 10-year-olds from 28.8% to 29.3%.^3^ This underscores the sustained academic significance and imperative of addressing the multifaceted issue of smoking globally.

 Jambi Province stands out with a notably high prevalence of smoking. According to data from the Central Statistics Agency (BPS) of Jambi Province, the number of active smokers has increased from 2015 to 2019. Data for Muaro Jambi Regency reveals that 21.44% are daily smokers, while 2.83% smoke occasionally.^4^ Research by Ablah et al indicates that rural and small-scale entrepreneurs lack tobacco-free policies compared to larger urban enterprises.^5^

 The implementation of smoke-free area policies holds considerable sway over community behavior, exerting a substantial influence on efforts to diminish or cease smoking habits. Insights from Philpot et al underscore this assertion, indicating that 11.5% of smokers are willing to quit upon encountering stringent enforcement of smoke-free policies, without a marked reduction in this inclination over time.^6^ Furthermore, Septiono et al corroborate this notion by establishing a tangible correlation between the execution of local smoke-free policies in Indonesia and a discernible decline in adult smoking intensity.^7^ This linkage underscores the pivotal role of localized interventions, such as establishing smoke-free zones, in safeguarding non-smokers from the deleterious effects of secondhand smoke.^8^

 The regulatory landscape in Muaro Jambi Regency reflects a concerted effort to address tobacco-related concerns legislatively. Beginning with Regional Regulation No. 5 of 2018 about Smoke-Free Areas, the local administration has demonstrated a commitment to fostering smoke-free environments within the region. Despite the promulgation of these regulations, empirical observations reveal a persistent prevalence of smoking violations, particularly within the precincts of healthcare facilities.^9,10^ This disjuncture between regulatory intent and observed practice prompts an inquiry into the efficacy of extant policies in curbing smoking behavior within healthcare services in the Muaro Jambi Regency.

 The proliferation of smoking infractions within healthcare settings warrants an examination of the underlying factors impeding the effective implementation of smoke-free policies.^11,12^ Firstly, inherent challenges in enforcement mechanisms may hinder the rigorous imposition of regulatory stipulations, necessitating a reevaluation of enforcement strategies and resource allocation. Additionally, the prevalence of smoking within healthcare facilities may stem from entrenched social norms or cultural practices that mitigate compliance with smoke-free mandates. Addressing these sociocultural determinants demands a multifaceted approach encompassing community engagement, public awareness campaigns, and targeted behavioral interventions.^13^

 Furthermore, institutional factors, such as a lack of clear guidelines or protocols for smoke-free compliance monitoring, may contribute to lapses in enforcement efforts.^14^ Inadequate training or awareness among healthcare personnel regarding their roles and responsibilities in upholding smoke-free policies could also exacerbate compliance challenges. Thus, bolstering capacity-building initiatives and fostering a culture of compliance within healthcare institutions emerge as imperative strategies to fortify smoke-free initiatives.

 Moreover, the persistent prevalence of smoking infractions within healthcare facilities underscores broader systemic deficiencies in governance, enforcement, and accountability mechanisms. Effective policy implementation necessitates a coordinated and collaborative approach involving stakeholders, including policymakers, regulatory agencies, healthcare providers, and civil society actors.^15,16^

 The rationale for adopting a mixed-methods design in this study is rooted in the complexity of addressing smoke-free policy compliance within healthcare settings. While quantitative methods offer the advantage of systematically measuring non-compliance prevalence and identifying correlating factors, they often fall short of capturing the nuanced, contextual dynamics influencing these patterns. Qualitative approaches provide a deeper understanding of the underlying motivations, perceptions, and institutional barriers that may not be readily apparent through numerical data alone. Thus, integrating quantitative and qualitative methods allows for a more comprehensive exploration of the phenomenon, aligning with the study’s aim to generate actionable insights for policy improvement. This study aims to evaluate the implementation of smoke-free zones in healthcare services. Strengthening intersectoral collaboration and institutional coordination is indispensable for enhancing the efficacy and impact of smoke-free initiatives in the Muaro Jambi Regency and beyond, making this evaluation crucial for identifying areas of improvement and policy enhancement.

## Method

###  Research design

 This study employs a convergent mixed-methods design, combining quantitative data for generalizability and qualitative data for contextual depth, enabling comprehensive analysis, triangulation, and deeper insights into smoke-free zone implementation in healthcare settings. In the quantitative aspect, a cross sectional survey based on nine criteria for Smoke-Free Zones is conducted to assess spatial distribution.^17,18^ The criteria include: 1) the presence of anti-smoking campaigns or signage; 2) the absence of tobacco product vendors; 3) the absence of individuals actively smoking; 4) the absence of cigarette advertisements; 5) the absence of the smell of cigarette smoke; 6) the absence of ashtrays; 7) the absence of designated areas for smoking; 8) the absence of vendors selling electronic cigarettes; and 9) the absence of individuals using electronic cigarettes.

 The qualitative component involves one-on-one interviews with informants, exploring the implementation of smoke-free policies in healthcare centers, and considering factors such as compliance, designated areas, enforcement, anti-smoking media, absence of vendors/advertisements, and overall awareness.^17^ Informants were interviewed about the condition of smoke-free zones within healthcare centers and the implementation of policies regulated by government and regional laws. The interviews explored key questions such as “Have there been any official announcements regarding the implementation of smoke-free zones?” and “How is information about smoke-free zone regulations communicated to the community?” The data collection spanned six months, starting in June until December 2023, and was conducted at various healthcare facilities in Muaro Jambi, Indonesia.

###  Study setting and population

 The study was conducted in Muaro Jambi Regency, a district in Jambi Province, Indonesia. This region was selected due to its implementation of Regional Regulation No. 5 of 2018 concerning Smoke-Free Areas, which provided a legislative framework for smoke-free policies in healthcare facilities. The study population for the quantitative component comprised all healthcare facilities in Muaro Jambi Regency, including hospitals, community health centers (Puskesmas), and clinics (Pustu). For the qualitative component, the population included healthcare facility staff, administrators, and regional policymakers involved in smoke-free policy implementation.

###  Sampling technique and sample size

####  Quantitative component

 A total of 74 healthcare facilities were surveyed using stratified random sampling to ensure representing across different facility types in every district in Muaro Jambi Regency. This sampling approach ensured that all types of healthcare facilities (hospitals, community health centers, and clinics) across all districts within the regency were included, providing a complete spatial representation of smoke-free policy implementation.

####  Qualitative component

 For the qualitative phase, purposive sampling was employed to select 31 informants (Table 1). Participants were strategically chosen to include perspectives from healthcare facility leadership, staff, and policymakers at various administrative levels. Selection criteria included: 1) direct involvement in smoke-free policy implementation or enforcement; 2) minimum of one year working in their current position; and 3) willingness to participate in in-depth interviews. The sample size was determined by data saturation, where interviews continued until no new themes emerged.

**Table 1:** Research informants

**Institution**	**Status**	**Age (year)**	**Gender**	**Code**
Kindergarten (PAUD)	Teacher	50	Female	SL.06
	Student's Parent	29	Female	SL.27
Elementary School (SD)	Staff	35	Male	SL 02
	Headmaster	37	Male	SL 13
	Headmaster	59	Male	SL 14
	Headmaster	45	Male	SL 15
	Teacher	37	Male	SL16
Islamic Elementary School (MI)	Teacher	51	Female	SL.18
Junior High School (SMP)	Teacher	37	Male	SL 04
	Visitor	19	Male	SL.24
	Teacher	36	Male	SL.25
	Teacher	41	Male	SL26
Islamic Junior High School (MTs)	Teacher	28	Female	SL.03
	Teacher	34	Female	SL.09
	Teacher	40	Male	SL.10
	Headmaster	46	Male	SL 11
Senior High School (SMA)	Headmaster	40	Female	SL.20
	Visitor	17	Female	SL.21
	Teacher	40	Male	SL.22
Vocational High School (SMK)	Teacher	35	Male	SL.23
Islamic Senior High School (MA)	Student	15	Female	SL.05
	Teacher	55	Female	SL.07
	Staff	35	Male	SL.23
Higher Education (University)	Lecturer	40	Male	SL.30
	Visitor	35	Male	SL.17
Education Department (Dinas Pendidikan)	Chief	48	Male	SH.03
Regional Police Force (Satpol PP)	Chief	50	Male	SH.06
Regional Legislative Council (DPRD)	Vice of Chief	51	Male	SH.08
Ministry of Religious Affairs in Muaro Jambi Regency	Chief	51	Male	SH.07
Indonesian Ulama Council in Muaro Jambi Regency (MUI)	Chief	59	Male	SH.02
Health Department (Dinas Kesehatan)	Chief	49	Male	SH.09
Total	31 Informants			

###  Data collection instruments and procedures

 Data collection for the spatial survey utilized KoboToolbox, an open-source Android-based application. A structured observation checklist based on the nine criteria for Smoke-Free Zones was developed and validated through expert review and pilot testing before implementation. Twenty-two trained enumerators conducted on-site observations at each healthcare facility, recording GPS coordinates and compliance with each criterion. The enumerators underwent standardized training to ensure consistent application of the assessment criteria across all facilities.

 In-depth interviews were conducted using a semi-structured interview guide developed based on literature review and expert consultation. The guide explored key dimensions of smoke-free policy implementation, including leadership commitment, enforcement mechanisms, perceived barriers, facilitators, and recommendations for improvement. Interviews were conducted by trained research assistants in Indonesian language, audio-recorded with permission, and lasted 45-60 minutes each. Interviews took place in private settings at the participants’ workplaces to ensure confidentiality and comfort.

###  Data analysis

 Data from the spatial survey were initially cleaned and validated before analysis. Descriptive statistics, including frequencies and percentages, were calculated using SPSS 16.0 to summarize the prevalence of compliance with each smoke-free criterion and overall compliance across different types of healthcare facilities.^19^ Cross-tabulation analysis was performed to examine relationships between facility type and compliance status. Spatial mapping of smoke-free zone implementation was conducted using Quantum Geographic Information System (QGIS) 3.30.2 to visualize geographical patterns of compliance across the regency.^20^

 Audio recordings of interviews were transcribed verbatim and analyzed using conventional content analysis following Bengtsson’s methodology.^21-24^ The analysis process involved: 1) familiarization with data through repeated reading of transcripts; 2) systematic coding of text segments; 3) categorization of codes into meaningful clusters; 4) abstraction into themes and sub-themes; and 5) interpretation of findings. Two researchers independently coded the data to enhance analytical rigor, with discrepancies resolved through discussion until consensus was reached. The analysis focused on identifying barriers and facilitators to smoke-free policy implementation, as well as contextual factors influencing compliance.

###  Integration of findings

 Following the convergent mixed-methods design, quantitative and qualitative findings were integrated at the interpretation stage. This integration involved comparing and contrasting findings from both components to identify areas of convergence, divergence, and complementarity. The integrated analysis provided a comprehensive understanding of the implementation status of smoke-free policies in healthcare facilities and the underlying factors influencing compliance patterns.

###  Ethical considerations

 The study protocol received ethical approval from the Health Polytechnic Ministry of Health Jambi (Poltekkes Kemenkes Jambi) (Ethical Permit: LB.02.06/2/645/2022). All participants provided written informed consent before participation. Confidentiality was maintained by using codes instead of names in all data records. Participants were informed of their right to withdraw from the study at any time without consequences. All procedures were conducted in accordance with the ethical standards of the responsible committee on human experimentation and with the Helsinki Declaration.

## Results

###  Regulation Implementation

 The implementation of Regional Regulation No. 5 of 2018 concerning Smoke-Free Areas in the educational, healthcare, worship, and children’s play areas in Muaro Jambi Regency is facing several challenges. According to the informant, there is no registered public transportation in the Muaro Jambi Regency Transportation Agency. Despite establishing this regulation, the execution of Smoke-Free Areas has not been optimal.

 Consistent socialization regarding Smoke-Free Areas has not been conducted, leading to suboptimal implementation. Interview results indicate that the socialization of this regulation was only carried out in the early stages when it was enacted. Following the enactment of Regional Regulation No. 5 of 2018, socialization occurred through meetings with Regional Apparatus Organizations (OPD) and sending letters containing information about the regulation to OPD. However, this socialization program has not been consistently continued up to the present, as explained by the informant:

 “*If we’re talking about official announcements, they haven’t been scheduled yet. However, when folks from the health center drop by, they talk to us about it. It’s just not all structured and planned out” *(SL. 11).

 “*Yeah, it’s like, if the legal team is specifically doing a session on the no-smoking zone regulations, there’s... well, it did happen before. But, you know, it’s not something they schedule regularly. It’s more like a once-a-year thing; budget-wise, it’s tight, making it tricky” *(RS.03).

###  Healthcare facilities

 In-depth interviews on the impact of the Smoke-Free Areas policy in healthcare facilities revealed that many facilities do not adhere to it. According to the information obtained, the rural community finds it challenging to comprehend the prohibition of smoking within healthcare facilities. There is a discrepancy in perceptions regarding the no-smoking rule. At the same time, it is expected not to smoke within hospitals, health centers, and clinics; the perception among staff and the community is that smoking is allowed outside the buildings.

 The interview results found that the general public generally agrees that smoking is prohibited in healthcare facilities. In practice, if someone is found smoking within the hospital premises, no sanctions or other penalties are imposed according to the local regulations. Prevention efforts are limited to verbal warnings. Observations also revealed that both healthcare professionals and employees are still found smoking within the facility premises or inside the building.

 In-depth interviews uncovered that officials in healthcare services engage in smoking activities within their working spaces, citing difficulty in quitting smoking. This behavior has an impact on visitors who then engage in smoking behavior within the healthcare facility. The informant conveyed this information as follows:

 “*So, the health center in Sungai Duren has a policy that prohibits employees who smoke from lighting up indoors. They can smoke outside, but it must be after work hours and not in areas where services are provided” *(PKM.11).

 From in-depth observation of healthcare facilities, including community health centers, health posts, and clinics, it was found that almost all do not implement Smoke-Free Areas that displayed in Table 2.

**Table 2:** Observation results for smoke-free and non-smoke-free areas in healthcare facilities in Muaro Jambi Regency

**No.**	**Variable**	**n**	**%**
1.	No smoking area		
	Compliant	19	25.7
	Non-compliant	55	74.3
2.	Type of health institutions		
	Healthcare Center	22	29.7
	Hospital	3	4.1
	Clinic (Pustu)	49	66.2
3.	District		
	Bahar Selatan	1	1.4
	Bahar Utara	3	4.1
	Jambi Luar Kota	17	23.0
	Kumpeh	14	18.9
	Kumpeh Ulu	3	4.1
	Maro Sebo	1	1.4
	Mestong	11	14.9
	Sekarnan	11	14.9
	Sungai Bahar	8	10.8
	Sungai Gelam	4	5.4
	Taman Rajo	1	1.4
4.	Criteria for smoke-free zones		
	There is media prohibition of smoking		
	Yes	22	29.7
	Not	52	07.3
	There is a cigarette seller		
	Yes	27	36.5
	Not	47	63.5
	There are people smoking		
	Yes	17	23.0
	Not	57	77.0
	There are cigarette ads		
	Yes	10	14.1
	Not	64	85.9
	There is a smell of smoke		
	Yes	15	20.3
	Not	59	79.7
	There is an ashtray.		
	Yes	22	29.7
	Not	52	70.3
	There is a smoking area.		
	Yes	8	10.8
	Not	66	89.2
	There are e-cigarette sellers		
	Yes	1	1.4
	Not	73	98.6
	There are e-cigarette smokers		
	Yes	6	8.1
	Not	68	91.9

Source: Data Analysis (2022).

Table 2 indicates that most healthcare institutions, amounting to 74.3%, do not implement smoke- free areas (Kawasan Tanpa Rokok - KTR). The most prevalent type of healthcare facility is clinics and health posts (Pustu), constituting 66.2% of the observed institutions. The highest frequency of observations occurred in Jambi Luar Kota district, accounting for 23%. This distribution highlights the primary locations where smoke-free zone policies are being implemented and assessed. Understanding the types and locations of healthcare facilities is crucial for evaluating the effectiveness of these regulations, as different facility types may face unique challenges in enforcing smoke-free policies (Table 3).

**Table 3:** Cross-tabulation table of healthcare facility types with smoke-free area categories in Muaro Jambi Regency for 2022

**Type of health facility**	**Implementation of non-smoking areas**				**Total**	
	**No**		**Yes**			
	**n**	**%**	**n**	**%**	**n**	**%**
Healthcare center	22	100	0	0	22	100
Hospital	3	100	0	0	3	100
Clinic (Pustu)	30	61.2	19	38.8	49	100
Total	55	74.3	19	25.7	74	100

**Figure 1:**
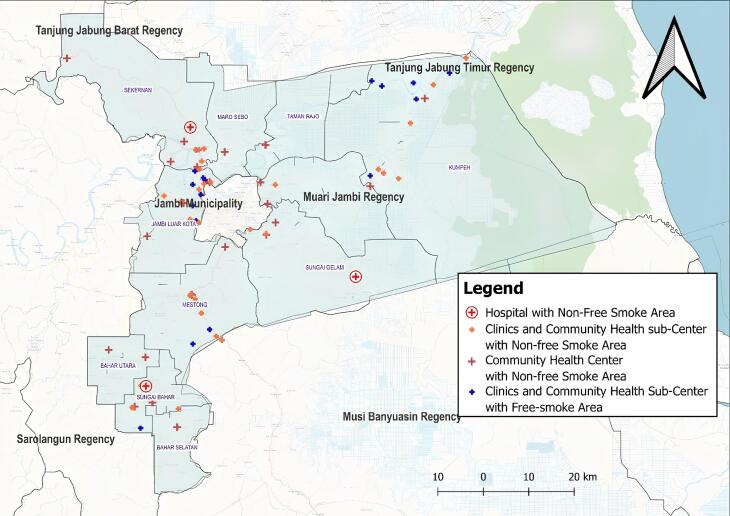
Health service institutions implementing smoke-free areas and not implementing smoke-free areas in Muaro Jambi Regency in 2022

 The above cross-tabulation table indicates that the most dominant health facilities that are not implementing smoke-free areas (KTR) are health centers (puskesmas) and hospitals, each registering 100%. This finding suggests significant challenges in enforcing smoke-free regulations within larger healthcare institutions, which may be attributed to higher patient and visitor traffic, limited supervision, or inadequate enforcement mechanisms. Figure 1 further illustrates the distribution of health service institutions that have implemented and those that have not implemented smoke-free areas across Muaro Jambi Regency in 2022, providing a visual representation of compliance levels and highlighting areas requiring stronger policy enforcement.

## Discussion

 Regional Regulation on Smoke-Free Areas in Muaro Jambi Regency was created based on the need for tobacco control and the importance of maintaining air quality in public services. The regulation involved various sectors and followed the procedures for drafting regional regulations. Proponents and opponents emerged during the formation of this regulation due to stakeholders involved in smoking behavior. Smoke-free policies impact smoking cessation efforts and prevalence.^25^ Through policies like establishing smoke-free areas or raising tobacco taxes, tobacco control has proven effective in reducing smoking prevalence.^14^ This study highlights the complex interplay of multi-level factors influencing policy implementation, informing strategies for optimizing smoke-free policies in residential areas.^26^ In Uganda, the study reveals intrinsic challenges in developing a comprehensive government approach, indicating significant uncertainty and ambiguity among decision-makers regarding tobacco control governance.^27^ The strength of tobacco control policies as public interventions can influence substantial changes in cultural norms within public health services.^28^

 Regional Regulation No. 5 of 2018 involved various stakeholders, including the Health Department, Education Department, Transportation Department, and the Regional Council of Islamic Scholars in Muaro Jambi Regency. This collaboration aimed to gather input and perspectives from stakeholders, resulting in policies that promote tobacco control in the region. National and local advocates working with researchers and lawyers can influence effective tobacco control policies.^29^ Tax policies on tobacco excise positively impact changes in tobacco consumption patterns.^30^ Prohibiting the use of long-standing tobacco products like smoking is often culturally challenging for legislators due to concerns about public reactions.^31^

 Program Implementation From the interview results, this regional regulation seems to experience negligence in its implementation. There is a lack of sustained socialization, and stakeholders do not continue efforts to enforce the enacted regulation, as evidenced by the absence of derivative regulations outlined in Regional Regulation No. 5 of 2018. Smoking bans in workplaces positively impact employee health.^32^ Implementation in Healthcare Services Healthcare services, as the focal point for implementing smoke-free areas, have not fully adhered to the regulation. The implementation is limited to a small portion, confined within the building, while the expectation from the regulation and other rules is that smoke-free areas should be applied throughout the healthcare environment, including health centers, hospitals, and clinics in Muaro Jambi Regency. The main barriers and facilitators for tobacco cessation in the population in the context of group behavior counseling interventions need examination.^33^ For healthcare workers, the information system should identify smokers, advise them to quit tobacco use, and follow up with them even after they leave the healthcare facility.^34^

 Healthcare providers and staff play a pivotal role in shaping the culture and environment within healthcare facilities, extending beyond their clinical duties to encompass advocacy for public health initiatives such as tobacco control.^35^ As custodians of health and wellness, they exemplify healthy behaviors, setting a standard for patients, visitors, and the broader community. In the context of tobacco control, their actions and attitudes towards smoking directly influence perceptions and behaviors surrounding tobacco use within healthcare settings. Thus, leveraging their influence to discourage smoking aligns not only with ethical imperatives but also with the objectives of regional regulations to promote public health.^36^

 By the principles of medical ethics, healthcare providers are duty-bound to prioritize the well-being of their patients and the community they serve. Central to this obligation is promoting health-enhancing behaviors and preventing harm, including addressing tobacco use’s detrimental effects. By abstaining from smoking and actively advocating against it, healthcare professionals uphold their commitment to beneficence and non-maleficence, fulfilling their moral obligations to promote the greater good.

 Moreover, healthcare providers possess a unique platform to disseminate health information and influence behavior change. Patients and visitors often view them as trusted sources of guidance and expertise, lending credibility to their messages regarding tobacco cessation and avoidance.^37^ Through targeted interventions, such as brief counseling sessions or the distribution of educational materials, healthcare professionals can empower individuals to make informed decisions about their tobacco use and take proactive steps towards cessation. By harnessing their position of authority and trust, they can amplify the impact of tobacco control efforts, catalyzing positive health outcomes at both the individual and population levels.

 Compliance with regional regulations about smoke-free healthcare facilities is not merely a legal obligation but a moral imperative rooted in social responsibility and professional ethics.^38^ By adhering to these regulations, healthcare providers demonstrate their commitment to upholding public health standards and promoting the common good. Moreover, they contribute to the broader societal efforts to denormalize smoking and reduce its prevalence, thereby mitigating the burden of tobacco-related diseases and improving population health outcomes.

 Healthcare providers and staff occupy a privileged position as agents of change within healthcare settings, capable of exerting considerable influence on tobacco-related behaviors and attitudes. Embodying the principles of health promotion, advocacy, and ethical practice, they can serve as powerful catalysts for tobacco control initiatives, fostering environments prioritizing health and well-being.^8,39^ Through their concerted efforts, healthcare facilities can become beacons of health, resilience, and empowerment, inspiring individuals to embrace tobacco-free lifestyles and realize their full potential for health and vitality.

## Conclusion

 The effectiveness of smoke-free area policies in healthcare services hinges on local government entities’ steadfast oversight and commitment. Leaders and staff within healthcare settings serve as exemplars, reinforcing compliance with smoke-free regulations through their actions. Policy interventions, notably Regional Regulations, are instrumental in tobacco control efforts. However, their efficacy is contingent upon the unwavering support of decision-makers, particularly local leaders, and the consistent dedication of healthcare organizational leaders. The absence of endorsement from these influential figures may undermine the impact of policies on smoking behavior control within healthcare facilities. Therefore, sustained vigilance and involvement from decision-makers and relevant stakeholders are imperative to ensure continual enforcement and effectiveness of smoke-free area policies.

## Competing Interests

 The authors declare no competing interests.

## Ethical Approval

 The study protocol was approved by the Health Polytechnic Ministry of Health Jambi (Poltekkes Kemenkes Jambi) (Ethical Permit: LB.02.06/2/645/2022).
